# Early *Versus* Late Removal of Internally Fixated Kirschner’s Wires for Displaced Lateral Condyle Fracture of Humerus in Children

**DOI:** 10.2174/1874325001812010229

**Published:** 2018-06-29

**Authors:** Hari Prasad Sapkota, Poojan K Rokaya, Mangal Rawal, Dhan Bahadur Karki, Deoman Limbu

**Affiliations:** 1 Department of Orthopedics, Mid-Western Regional Hospital, Surkhet, Nepal; 2 Department of Orthopedics, Karnali Academy of Health Sciences, Jumla, Nepal

**Keywords:** Early, Internal Fixation, Kirschner’s Wire, Lateral Condyle

## Abstract

**Introduction:**

Lateral condyle fracture of the distal humerus is the second most common paediatric elbow fracture. Unstable, rotated and displaced (>2 mm) fractures are managed with open reduction and internal fixation with Kirschner’s wires or screws. Debate persists as for how long the Kirschner’s wires should be placed in situ after internal fixation. We aimed to compare the functional and radiological outcome after early *versus* late removal of internally fixated Kirschner’s wires for displaced lateral condyle fracture of distal humerus.

**Methods:**

Children that underwent early (3-4 weeks) or late (5-7 weeks) removal of Kirschner’s wire after open reduction and internal fixation for displaced lateral condyle fracture of humerus were observed for a period of minimum 6 months. Time to radiological union, carrying angle, range of motion was assessed and compared between early and late group. Functional outcome was compared using the Dhillon scoring system.

**Results:**

We report the outcome of 40 cases (20 cases in each early and late group). Radiological union was achieved in all the cases of both group at 12 weeks follow up. The mean loss of carrying angle was statistically insignificant (*p* = 0.394) between the early and late group. There was no significant difference between the early and late group in relation to arc of motion at 12 weeks (*p*=0.724) and 6 months (*p*=0.638) follow up. Using the Dhillon scoring system, there was 100% excellent Dhillon score in early group, 80% excellent and 20% good Dhillon score in late group. Functional outcome was statistically insignificant between the two groups (*p* = 0.106)

**Conclusion:**

Early removal of internally fixated K-wires for displaced lateral condyle fracture of humerus in children showed similar radiological and functional results to late removal.

## 1 INTRODUCTION

Lateral condyle fracture of the distal humerus is the second most common paediatric elbow fracture after supracondylar humeral fractures [1]. It represents approximately 17% of all distal humerus fractures in paediatric population [2]. They usually occur as the result of a fall onto outstretched hand with the elbow in full extension, forearm in supination with forced varus angulation. Functional loss of Range Of Motion (ROM) is common with displaced lateral condyle fracture of humerus because the fracture extends into the joint surface. Surgical corrections of malunited lateral condyle fracture have a poor response as compared to correction of malunited supracondylar fracture of humerus in children [3]. Potential complication like nonunion, cubitus varus, cubitus valgus, fishtail deformity, tardy ulnar nerve palsy, elbow joint stiffness make lateral condyle fracture a serious injury. Thus, the term “the fracture of necessity”

Various classification systems have been adopted to classify this fracture. The Milch classification describes the anatomic location of the fracture and its relation to the capitellotrochlear groove of the distal humerus [4]. Milch type I fracture passes lateral to the trochlea into the capitellotrochlear groove whereas Milch type II fracture extends into the apex of trochlea. Based on the degree of displacement Jakob described three staged classification [5]. Stage I fracture is non-displaced with intact articular hinge. Stage II the fracture extends through the articular surface with translation and angulation. Stage III condylar fragment is rotated and totally displaced laterally and proximally. Minimally displaced fractures (<2 mm) are managed in long arm posterior slab with serial check x-rays. Closed reduction and percutaneous pinning has become a viable option for limited initially displaced fractures and fractures with intact articular hinge [6, 7]. However to avoid complications early accurate reduction is desired with stable fixation. Unstable, rotated and displaced (>2 mm) fractures are managed with Open Reduction and Internal Fixation (ORIF) with Kirschner’s (K) wires or screws. Debate persists as for how long the K wires should be placed in situ after internal fixation of displaced lateral condyle fracture of humerus. The time of K wires removal in the available literature ranges from 3 to 8 weeks [8, 9]. The purpose of this study is to assess and compare the functional and radiological outcome after early *>versus* late removal of internally fixated Kirschner wires for displaced lateral condylar fracture of humerus in children.

## 2 MATERIALS AND METHODS

A prospective comparative study was conducted among 40 children (early 20, late 20) at Karnali Academy of Health sciences Jumla, Nepal from July 2014 to June 2017 after ethical approval. We report only those patients in whom displaced lateral condyle fracture of distal humerus were stabilized solely using K wires with a inclusion criteria of age 4-14 years, displaced and grossly rotated fracture, patients with adequate follow-up and complete medical records. Patients with open fracture, old fracture, established elbow deformity prior to fracture, associated injury in the same limb, fracture with neurovascular injury, pathological fractures were excluded from the study. Patient’s age, sex, side, mode of injury, time to surgery, time of radiological union, Carrying Angle, ROM were evaluated at subsequent follow up after taking an informed written consent. No extra financial burden was given to the patients. Initially, all the fractures were immobilized in long arm posterior slab and the patients were advised to elevate the affected limb with gentle ROM of fingers. Radiographic evaluation was done with standard antero-posterior and lateral views of the injured elbow to classify the fracture as per Milch classification. All the patients underwent ORIF *via* lateral ‘J’ Kocher’s incision. Two parallel or divergent K wires of 1.5 to 2mm size were used percutaneously for fracture fixation. Most of the patients were discharged on the 2^nd^ postoperative day after wound inspection and check x-ray. First follow – up was on second postoperative week, on which wound inspection, pin tract dressing and suture removed. Second follow - up was on third postoperative week, on which all cases were randomly divided into two groups;

Early Group: all those patients whose K wires and above elbow plaster slab was removed mainly on the 3^rd^ postoperative week. Removal of K wires and above elbow slab on 4^th^ postoperative weeks were also included in this group.

Late Group: Late group was defined as that in which removal of K wires and above elbow posterior slab was done mainly on six postoperative weeks but removal of K wires and above elbow posterior slab on 5^th^ and 7^th^ postoperative weeks were also included.

In all early group cases, on third week postoperatively K wires and above elbow posterior slab were removed. Dressing was done. ROM of the elbow was evaluated. Check x-ray was taken and gentle protected active ROM exercise commenced. The patients were advised to continue physiotherapy at home regularly. In all late group cases on third week post operatively - pin tract dressing was done and check x-ray was taken. The K wire and above elbow plaster slab were continued for 6 weeks. Third follow - up was on 6^th^ week postoperatively. In early group - ROM and carrying angle of the injured limb was measured. Check x-ray was taken to see the callus. In late group K wire and slab were removed. The ROM of the elbow and carrying angle were measured and check x-ray was taken. Active ROM was started. Fourth follow - up was on 12^th^ postoperative week on which ROM carrying angle of injured and healthy limbs of both early and late groups were measured and check x - ray of the injured elbow were taken to evaluate union or nonunion. Radiological union was defined as the appearance of bridging callus at the fracture site on both the planes. Fifth follow - up was on 6^th^ postoperative month on which final evaluation was done as per the Dhillon scoring system (Table **1**) [10]. Carrying angle and ROM was measured with goniometer using the standard technique. Every measurement was taken twice by the orthopaedic surgeon to ensure accuracy. Data analysis was done using Statistical Package for Social Sciences (SPSS Inc. version 17, Chicago, Illinois). Fisher exact test and Independent *t*-tests were used for the comparison of data. The significance (*p*) was set below 0.05.

## 3 RESULTS

We report the outcome of 40 patients with complete medical records. There were 20 cases in each early and late group. The demographic data including age, sex, side, mode of injury, fracture type and time to surgery are tabulated in Table **2**. The mean time of pin and plaster removal in early group was 3.2 weeks (range 3-4 weeks) whereas 5.85 weeks (range 5-7 weeks) in late group. Radiological union was achieved in all the cases of both the early and late group at 12 weeks follow up. Statistically, fractures united without significant difference in early and late groups at 12 weeks (*p* = 1.00). Carrying angle of healthy and injured limb of all the patients in both the early and late group are mentioned in Table **3**. The mean loss of carrying angle in early group was 1.3±1.03° whereas 1.9±0.9° in the late group. We did not find any statistical significant difference between early and late groups in relation to loss of carrying angle (*p* = 0.394) The ROM of healthy and injured limb in both the early and late group at 12 weeks and 6 months follow up are shown in Table **4**. No significant difference between early and late groups in relation to ROM was seen at 12 weeks (*p*=0.724) and 6 months (*p*=0.638). The mean loss of ROM in the early and late group was 0.85±1.84° and 2.6±2.01° respectively at 6 months follow up which was statistically insignificant (*p* = 0.542) Using criteria of Dhillon, there was 100% (20 cases) excellent Dhillon score in early group. In late group, there were 80% (16 cases) excellent Dhillon score and 20% (4 cases) good Dhillon score. No fair and poor Dhillon score were found in both groups (Table **5**). The functional outcome difference was found to be statistically insignificant between the two groups (*p* = 0.106)

## 4 DISCUSSION

Lateral humeral condyle fracture is the second most common fracture around the elbow after supracondylar fracture of the humerus [11]. The natural history and outcome of the acute fracture of the lateral condyle has been extensively studied but there are limited studies and information about the time of K wires removal. There is controversy as to whether fractures of the lateral humeral condyle unite by early removal of internally fixated K wires and above elbow posterior slab. Küçükkaya *et al* advised to consider the patients age for determining the fixation period in their study on surgical management of displaced fractures of lateral humeral condyle in children where the removal time ranged from 3-5 weeks [12]. Thomas *et al*. in a case series of 104 patients concluded that 3 weeks of immobilization with K wires in situ is sufficient to achieve healing after open reduction and internal fixation in most of the displaced lateral condyle fracture of humerus [13]. Flyn and Richards noted that immobilization of at least 12 weeks was often necessary even for the lateral condyle fracture with minimal displacement [14]. Cardona *et al*. advocated about retaining the internally fixated K wires until the radiological evidence of healing on anteroposterior, lateral and oblique views which averaged about 6 weeks or more [15]. However, there is no consensus as to when the implants should be removed, with the recommended time ranging from 3 to 8 weeks.

In this study, age of the patient ranged from 4 to 11 years. The mean age in early group was 6.3 years (4 - 11 years) and in late groups 6.5 years (4 - 12 years). Male to Female ratio in this study was 3.45: 1, which nearly coincides with the study of Toh *et al*, with 3.5: 1 of male to female ratio [16]. Ratio of involvement of non - dominant (left) to dominant (right) extremity was 1.86: 1 (26 cases involving left and 14 cases involving right). This finding is similar with the study of Bast *et al*., who found non - dominant involvement to be 2.1 times the dominant extremity [17]. Fall on outstretched hand was the most common mechanism of injury in both the early and late group. According to Milch classification, 95% (38) cases were Milch type II and only 5% (2) cases were Milch type I lateral condyle fracture. As for the time to surgery from the day of injury is concerned, mean time for ORIF in early group was 6.5 days (3 days - 10 days) and in late group was 6.8 days (2 days - 21 days), which were not significantly different (*p*= 0.87) between two groups. Although mean time of surgery was nearly same in two groups but range of time was more in late group. We did not encounter wound or pin tract infections in any of the patients of both the group.

As for the internally fixed K wires and plaster removal is concerned, all cases were randomly divided into early and late groups. The mean time of plaster and pin removal in early group was 3.2 weeks (3 weeks - 80% and 4 weeks - 20%) and in late group was 5.85 weeks (5 weeks - 20%, 6 weeks - 75% and 7 weeks - 5%).

On radiological evaluation at 12 weeks post-surgery, all fractures in early and late groups were united (100%) which could reflect that three weeks of K wire fixation and above elbow posterior slab immobilization achieves healing in most displaced lateral condyle fracture of humerus. There was a single case of non-union in the study group of 104 children with 3 weeks of K-wires fixation conducted by Thomas *et al*., [13]. Boz *et al*., did not observe any non-union in 69 patients treated with open reduction and 4 weeks of K-wires fixation for displaced lateral condyle fractures of the humerus [18].

At 12 weeks post-surgery follow- up, the available mean arc of motion was 140.10±3.82° in early group and 137.15±5.34° in late group. No statically significant difference was found (*p* = 0.724). Similarly, at 6 months post-surgery follow- up, the mean arc of motion was 141.2±2.83° in early group and 138.85±3.89° in late group. Again there was no statistically significant difference (*p* = 0.638) between early and late group. Thomas *et al*. in their study found a mean arc of 137° in the injured elbows [13]. Boz *et al* revealed a mean ROM of 135° after open reduction and four weeks K-wire fixation for displaced lateral condyle fracture of humerus in 69 children [18].

The mean carrying angle at 6 month follow up post-surgery was 7.4±0.82° (Healthy = 8.7±1.17°) in early group and 7.6±0.99° (Healthy = 9.5±0.88° in the late group. However there was no significant loss of carrying angle between early and late group (*p* = 0.394) at 6 months of follow up. Thomas *et al* did not find significant difference between injured and uninjured elbows in relation to the mean carrying angle (*p* = 0.05) [13] In the study conducted by Boz *et al*., the mean carrying angle in the injured elbow was 8° and 7.8° in the healthy elbow which was not statistically significant [18]. In this study the mean carrying angle was within the normal range according to Criteria of Dhillon *et al*. [10]

Boz *et al*., used the Hardacre criteria [19], for the functional evaluation and found excellent results in 54 fractures (78.3%), and good in 15 fractures (21.7%) among 69 children treated with open reduction and four weeks K-wire fixation for displaced lateral condyle fracture of humerus. In the present study we used the Criteria of Dhillon *et al*., with 100% excellent Dhillon score in early group where as 80% excellent and 20% good Dhillon score in late group. Statistically there was no significant difference in relation to functional outcome (*p* = 0.10) among the early and late group.

## CONCLUSION

We could not recognize difference between early and late removal of internally fixated K wires for displaced lateral condyle fracture of humerus in children. There was no significant difference in the rate of fracture healing, functional outcomes or complications in terms of wound and pin tract infection. Early removal of the internally fixated Kirschner's wire and above elbow slab has the advantage of early movement of the elbow, wrist and less loss of school days for school going children.

Regarding displaced lateral condyle fracture of humerus in children we recommend that if the patients present earlier after injury, ORIF with K wires gives excellent results and can be safely carried out on emergency basis. Removal of K wires at 3 week post-surgery and protected active ROM yield excellent functional outcome.

Shorter duration of follow. up, small no of patients in both the groups is the limitations of the present study. Nonetheless, it will provide a data to the literature in making a strong valid recommendation with regards to the timing of implant removal for internally fixated lateral condyle fracture in children.

## Figures and Tables

**Table 1:** Dhillon scoring system for the outcome of fractures of the lateral humeral condyle in children (Functional grading points: excellent6, good 5, fair 4, poor < 4).

Function		Carrying Angle (degrees)	Score Points, each Column
Pain or Weakness	Range of Motion (degrees)		
Nil	0-140	Valgus 7-10	3
Occasional	>15-125	Valgus < 20 Varus < 0	2
After heavy work	>30-110	Valgus 20-30 Varus 0-15	1
With normal activity Motor or sensory loss	<30-110	Valgus > 30 Varus > 15	0

**Table 2:** Patient and fracture characteristic.

Variables	Early Group (20 patients)	Late Group (20 patients)
Age	Mean 6.3±2.3 years Range 4-11 years	Mean 6.47±2.02 years Range 4-12 years
Sex	Male 15 (75%) Female 5 (25%)	Male 16 (80%) Female 4 (20%)
Side of fracture	Left 12 (60%) Right 8 (40%)	Left 14 (70%) Right 6 (30%)
Mode of injury	Fall on outstretched hand 12 Fall from height 4 Sports 2 RTA 2	Fall on outstretched hand 14 Fall from height 4 Sports 1 Bicycle injury 1
Milch Type of fracture	Type I 1(5%) Type II 19 (95%)	Type I 1(5%) Type II 19 (95%)
Time to Surgery	6.5 Days Range 3-10 Days	6.8 Days Range 2-21 Days

**Table 3:** Carrying angle at 6 months follow up:

Group	Healthy	Injured	Loss of carrying angle	*p*-value
	Mean± SD(Degree)	Mean± SD(Degree)	Mean± SD(Degree)	
Early	8.7± 1.1743	7.4±0.8208	1.3±1.03	0.394
Late	9.5±0.8885	7.6±0.9947	1.9±0.9	

**Table 4:** Arc of motion at 12 week & 6 month follow up:

Group		Healthy Mean± SD (Degree)	Injured Mean± SD (Degree)	*p*-value
12 week	Early	142.05±2.187	140.1±3.82	0.724
	Late	141.45±2.87	137.15±5.34	
6 month	Early	142.05±2.187	141.2±2.83	0.638
	Late	141.45±2.87	138.85±3.89	

**Table 5:** Functional rating as per dhillon criteria:

Rating	Early	Late	*p*-value
Excellent	20 (100%)	16 (80%)	0.106
Good	0	4 (20%)	
Fair	0	0	
Poor	0	0	
Total	20 (100%)	20 (100%)	
